# Clinical Risk Factors and Outcomes of Spontaneous Hepatoblastoma Rupture in Children: A 10-Year Propensity Score-Matched Study

**DOI:** 10.3390/jcm15187102

**Published:** 2026-09-13

**Authors:** Yun Peng, Yaoxing Wu, Lin Ma, Chengxian Yang, Jing Chen, Bo Xiang

**Affiliations:** 1 Department of Pediatric Surgery, West China Hospital, Sichuan University, Chengdu 610041, China; 2024324025144@stu.scu.edu.cn (Y.P.);; 2 Department of Anesthesiology, Sichuan Cancer Hospital & Institute, Sichuan Cancer Center, University of Electronic Science and Technology of China, Chengdu 610042, China; 3 Department of Pediatric Surgery, Laboratory of Pediatric Surgery, West China Hospital, Sichuan University, Chengdu 610041, China

**Keywords:** hepatoblastoma, spontaneous rupture, child, outcomes, propensity score matching, risk factors

## Abstract

**Background:** Spontaneous rupture of hepatoblastoma is uncommon but potentially fatal in children. Published cohorts often combine spontaneous, traumatic, biopsy-related, and treatment-associated events. We aimed to identify clinical risk factors for spontaneous rupture and evaluate its prognostic impact. **Methods:** We retrospectively reviewed 106 children with pathologically confirmed hepatoblastoma treated at a tertiary center from July 2010 to July 2020. After excluding 10 with incomplete data or follow-up, 96 were eligible. Fourteen children who presented with spontaneous rupture were included in the rupture group. Eighty-two children without rupture were eligible as controls, and 42 were selected after 1:3 propensity score matching for age, sex, height, and weight. Subsequent analyses were restricted to the 1:3 propensity score-matched cohort. However, the matched-set identifier was not incorporated into the group comparisons, regression models, or survival analyses. Rupture was diagnosed by contrast-enhanced imaging and/or intraoperative findings. Logistic regression was used to identify independent risk factors. Exploratory two-predictor ridge regression and 2000-bootstrap internal validation were used to examine maximum tumor diameter and macrovascular invasion. Overall survival (OS) and event-free survival (EFS) were estimated using Kaplan–Meier analysis. **Results:** The rupture group had lower hemoglobin levels, larger tumors, more frequent bilobar disease, higher PRETEXT stage, and substantially more vascular invasion. Exploratory multivariable analysis suggested associations of maximum tumor diameter (OR 3.078, 95% CI 1.62–5.55) and macrovascular invasion (OR 13.521, 95% CI 1.16–148.23) with rupture. Given the small number of rupture events and the wide confidence interval for macrovascular invasion, these estimates should be interpreted cautiously and not as confirmation of independent risk factors. Macrovascular invasion was associated with rupture in crude analysis (odds ratio 8.00, 95% confidence interval 2.05–31.16) and the exploratory ridge model (adjusted odds ratio 2.89, 95% bootstrap confidence interval 1.27–7.93). The combined model incorporating maximum tumor diameter and macrovascular invasion showed an apparent area under the curve of 0.930 and an optimism-corrected area under the curve of 0.924. Children with ruptures had significantly worse OS and EFS than matched controls. **Conclusions:** Spontaneous rupture was associated with a clinically aggressive presentation characterized by substantially larger tumors, advanced local disease, and more frequent macrovascular invasion, and affected children had markedly lower OS and EFS estimates than matched controls. The results support tumor burden and macrovascular invasion as clinically relevant warning features that may help identify children requiring closer surveillance and early multidisciplinary planning.

## 1. Introduction

Hepatoblastoma (HB) is the most common primary malignant liver tumor in childhood and accounts for approximately 70–80% of pediatric hepatic malignancies [1]. Most cases occur during the first five years of life and commonly present with abdominal enlargement or a palpable mass, often accompanied by a markedly elevated serum alpha-fetoprotein level. Outcomes have improved through cisplatin-based chemotherapy, complete surgical resection, and liver transplantation in selected patients, but prognosis remains heterogeneous and is strongly influenced by pretreatment extent of disease, metastasis, vascular involvement, age, alpha-fetoprotein level, and response to therapy [2,3,4].

Tumor rupture at diagnosis is uncommon, with reported frequencies of approximately 5–16% across cooperative-group and institutional series [5,6]. The 2017 PRETEXT revision records rupture as the R annotation factor, alongside vascular involvement, multifocality, extrahepatic extension, and metastatic disease [7,8]. Rupture was treated as a high-risk criterion in the SIOPEL-3/4 protocols and was also identified as an adverse pretreatment feature in international CHIC analyses [3,4,5,9,10]. These classifications underscore its clinical importance but do not establish whether rupture independently determines outcome or primarily accompanies advanced tumor biology.

Clinically, rupture creates both an immediate hemorrhagic emergency and a longer-term oncologic challenge. Hemoperitoneum may cause severe anemia, coagulopathy, hemorrhagic shock, and early death, requiring rapid resuscitation and individualized hemorrhage control by embolization, surgery, or supportive care [6,10,11,12,13]. Intraperitoneal tumor spillage raises concern for peritoneal progression or relapse, whereas published series also show that durable survival and definitive resection or transplantation remain achievable after stabilization [5,6,14]. A recent cohort further associated preoperative rupture with early postoperative complications, emphasizing the need for coordinated oncologic, surgical, interventional-radiologic, anesthesia, and critical-care planning [15].

The available evidence is nevertheless limited and heterogeneous. Pondrom et al. described poor event-free survival and peritoneal progression or relapse in a multicenter cohort, whereas other single-center studies have reported more favorable outcomes [5,6]. Chang et al. identified a large primary tumor and vascular invasion as risk factors for rupture [10], and Hsu et al. found that absence of rupture was associated with longer event-free survival in a high-risk cohort [14]. However, most studies were small and retrospective, and some combined spontaneous events with traumatic, biopsy-related, or treatment-associated rupture. These distinct settings may differ in mechanism, accompanying tumor features, management, and prognosis, thereby limiting direct comparison and leaving the clinical phenotype of spontaneous pretreatment rupture incompletely defined.

We therefore conducted a propensity score-matched cohort study focused on spontaneous HB rupture before any tumor-directed intervention. Our objectives were to characterize its presentation and emergency management, compare tumor features with those of children without rupture, explore clinical features associated with rupture, and describe subsequent survival outcomes. By excluding rupture after biopsy, trauma, chemotherapy, or other tumor-directed procedures, we sought to reduce etiologic heterogeneity and provide clinically useful, hypothesis-generating evidence for earlier recognition and multidisciplinary planning.

## 2. Materials and Methods

### 2.1. Study Design, Setting, and Population

This retrospective single-center cohort study was conducted at West China Hospital, Sichuan University, a tertiary academic referral center in southwest China. Consecutive children with pathologically confirmed HB treated between July 2010 and July 2020 were screened. The report was prepared with reference to the Strengthening the Reporting of Observational Studies in Epidemiology (STROBE) recommendations [16].

**Figure 1:**
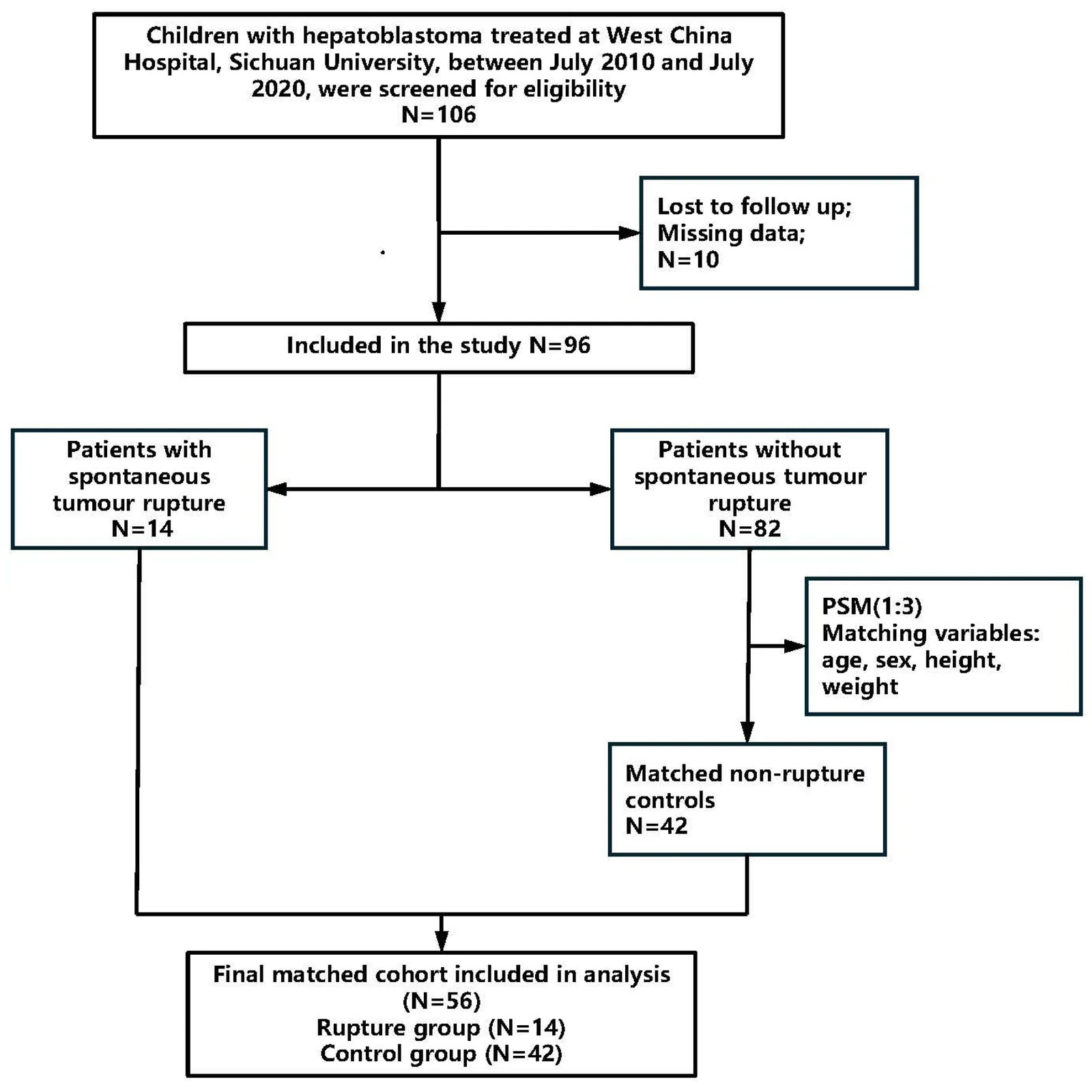
Patient selection and propensity score matching. HB, hepatoblastoma; PSM, propensity score matching.

Of the 106 children assessed for eligibility, 10 were excluded because clinicopathologic information or follow-up was incomplete. The complete-case cohort therefore comprised 96 children: 14 with spontaneous rupture and 82 without rupture. No statistical imputation was performed. The patient selection process is shown in Figure 1.

### 2.2. Definition and Diagnosis of Spontaneous Rupture

Spontaneous rupture was defined as rupture identified at initial presentation before biopsy, chemotherapy, embolization, ablation, or surgery for the index tumor. Children whose rupture occurred after abdominal trauma or after any tumor-directed procedure were not classified as spontaneous rupture cases.

**Figure 2:**
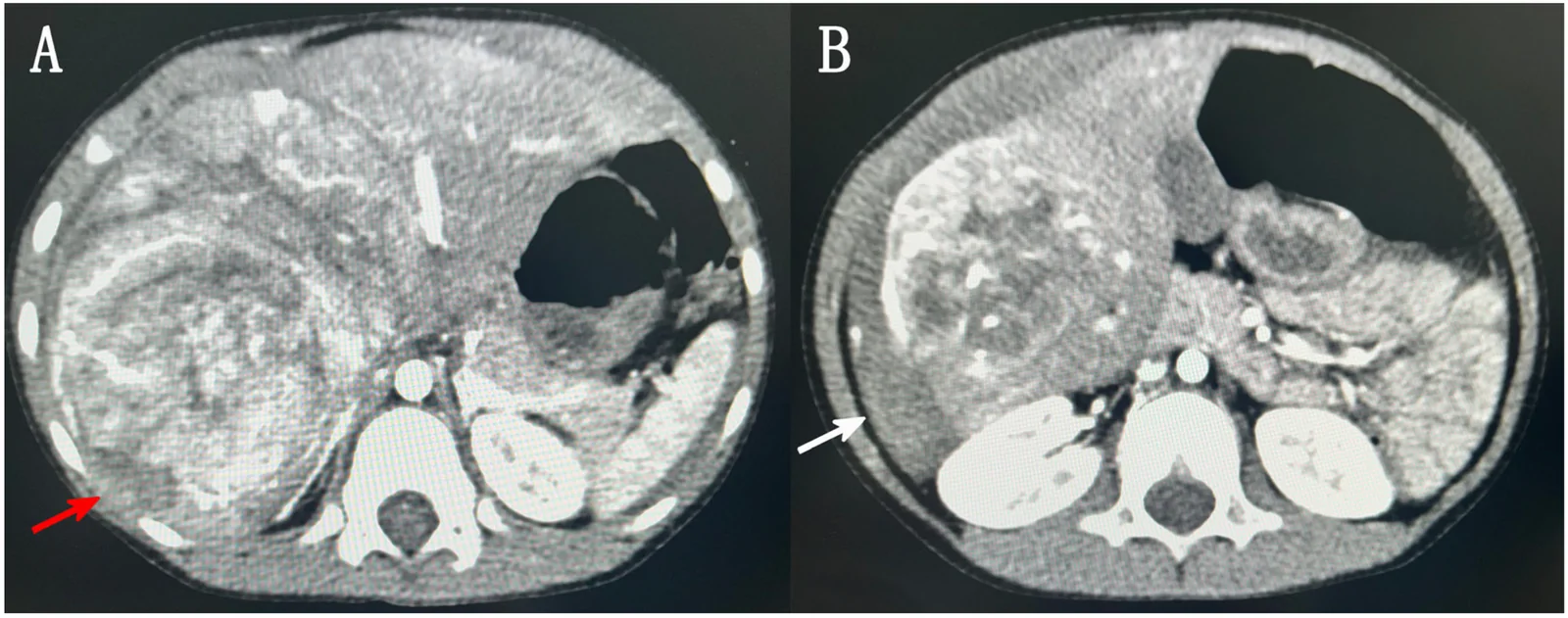
Contrast-enhanced computed tomography findings of spontaneous hepatoblastoma rupture: (**A**) Large hepatic mass with focal capsular discontinuity (red arrow). (**B**) Peritumoral and intraperitoneal fluid compatible with hemorrhage (white arrow).

The diagnosis was based on contrast-enhanced imaging and/or intraoperative findings, supported by clinical evidence of acute intra-abdominal hemorrhage when applicable. Imaging features included focal capsular discontinuity, a subcapsular hematoma or fluid collection, and intraperitoneal blood adjacent to the tumor. Representative computed tomography findings are shown in Figure 2.

### 2.3. Variables and Definitions

Demographic characteristics, presenting symptoms, laboratory measurements, imaging findings, histopathologic subtype, treatment, and follow-up outcomes were extracted from medical records. Laboratory values used in between-group comparisons were obtained at initial presentation before emergency intervention or chemotherapy. Tumor extent was categorized according to PRETEXT, and histopathologic subtype was classified according to the International Pediatric Liver Tumor Consensus Classification [17].

Macrovascular invasion was defined as radiologic encasement or obstruction of all three hepatic veins or the inferior vena cava, encasement or obstruction of both portal vein branches or the main portal trunk, or tumor thrombus in a major hepatic or portal venous branch [7].

Macrovascular invasion was analyzed separately from the PRETEXT stage because vascular involvement may vary within the same PRETEXT category and may plausibly contribute to congestion, tumor friability, and bleeding.

### 2.4. Emergency and Definitive Treatment Strategy

Children with rupture underwent immediate resuscitation, including rapid intravenous access, blood-product support, correction of coagulopathy, and close hemodynamic monitoring. The method of hemorrhage control was individualized according to circulatory stability, tumor anatomy, resectability, local expertise, and response to initial supportive care. If conservative management failed to stabilize the patient’s condition, prompt emergency intervention was undertaken, including emergency radical resection, partial hepatectomy for hemostasis, and transcatheter arterial embolization (TAE), an image-guided catheter-based procedure in which embolic material is delivered into the arterial supply to reduce or occlude blood flow and control hemorrhage [11,12,13,18,19]. In one exceptional salvage case after unsuccessful TAE, radiofrequency ablation (RFA), a local thermal-ablation technique that applies radiofrequency energy through an electrode to induce tissue coagulation and necrosis, was performed.

After stabilization, subsequent oncologic treatment was determined by a multidisciplinary team and was generally guided by the Expert Consensus for Multidisciplinary Management of Hepatoblastoma (CCCG-HB-2016), tumor extent, treatment response, and resectability [18]. Definitive treatment could include chemotherapy, delayed resection, or liver transplantation when indicated.

### 2.5. Follow-Up and Outcomes

Follow-up information was obtained from outpatient visits, inpatient records, and telephone interviews. Overall survival (OS) was defined as the interval from diagnosis to death from any cause or last follow-up. Event-free survival (EFS) was defined as the interval from diagnosis to relapse, progression, death, or last follow-up without an event. Complete response, relapse, and progressive disease were determined by multidisciplinary review of imaging, serum alpha-fetoprotein kinetics, operative findings, and pathology when available.

### 2.6. Propensity Score Matching and Statistical Analysis

Propensity scores were estimated from four variables specified before analysis: age, sex, height, and weight. These demographic and anthropometric variables were chosen because they were available before characterization of the index tumor and were intended to improve baseline demographic comparability. Tumor size, PRETEXT stage, bilobar involvement, and macrovascular invasion were not included in the propensity model because they were candidate tumor features under investigation and might represent markers on the pathway linking aggressive tumor biology with rupture. Nearest-neighbor matching without replacement was performed at a 1:3 ratio, using a caliper width of 0.05 times the standard deviation of the logit of the propensity score. Covariate balance was evaluated using standardized mean differences (SMDs), with values <0.10 considered acceptable.

Continuous variables are reported as median and interquartile range (IQR), except where otherwise stated, and were compared using the Mann–Whitney U test. Categorical variables were compared using the chi-square test or Fisher’s exact test, as appropriate. Given the limited number of rupture events, tumor location was simplified as unilobar versus bilobar disease and PRETEXT stage as I–II versus III–IV.

Variables with clinical relevance and significant between-group differences were entered into multivariable logistic regression. Because only 14 rupture events were available, association and prediction analyses were considered exploratory. A crude Fisher’s exact odds ratio was calculated for macrovascular invasion. A two-predictor L2 ridge-penalized logistic model was fitted with maximum tumor diameter as a continuous variable and macrovascular invasion as a binary variable; percentile-based 95% confidence intervals were estimated from 2000 bootstrap resamples. The matched-set structure was not incorporated into the regression. Model discrimination was quantified by the receiver operating characteristic area under the curve (AUC), and 2000 bootstrap resamples were used to estimate optimism and an optimism-corrected AUC. Calibration was assessed descriptively. The sensitivity and specificity reported for the archived operating point were calculated from the combined model’s predicted probabilities. Overall survival (OS) and event-free survival (EFS) were estimated by the Kaplan–Meier method and compared with the log-rank test. Two-sided *p*-values < 0.05 were considered statistically significant. Analyses were performed using R version 4.4.2 (R Foundation for Statistical Computing, Vienna, Austria) within RStudio IDE (Posit Software, PBC, Boston, MA, USA) and IBM SPSS Statistics version 27.0 (SPSS Inc., Chicago, IL, USA).

### 2.7. Ethical Considerations

The study was conducted in accordance with the Declaration of Helsinki and was approved by the Ethics Committee of West China Hospital, Sichuan University (protocol code 2023357). The requirement for informed consent was waived by the ethics committee because of the retrospective design and use of anonymized clinical data.

## 3. Results

### 3.1. Patient Selection and Matching

Among the 106 children screened, 96 had complete clinicopathologic and follow-up data and were eligible for analysis. Fourteen presented with spontaneous rupture and 82 did not. After 1:3 propensity score matching, 42 controls were retained, yielding a final matched cohort of 56 children. Age, sex, height, and weight were well balanced after matching, with all SMDs below 0.10 (Table S1).

### 3.2. Clinical Characteristics at Presentation

Compared with matched controls, children with rupture had lower hemoglobin at presentation (80.0 versus 103.0 g/L; *p* = 0.032), a significantly larger maximum tumor diameter (median, 14.00 versus 10.55 cm; *p* < 0.001), more frequent bilobar disease (92.9% versus 45.2%; Fisher *p* = 0.002), more advanced PRETEXT stage (III–IV in 92.9% versus 45.2%; Fisher *p* = 0.002), and more frequent macrovascular invasion (71.4% versus 23.8%; *p* = 0.003) (Table 1). Histopathologic subtype and the other measured laboratory variables did not differ materially between groups (Table S2).

### 3.3. Presentation, Emergency Management, and Oncologic Course

Among the 14 children with rupture, 8 were boys, and 6 were girls. The median age at presentation was 76.4 months. Thirteen children presented with sudden abdominal pain, whereas 1 initially presented with an abdominal mass and subsequently ruptured before tumor-directed treatment. All 14 had anemia, and four developed hemorrhagic shock. One child had a contained subcapsular rupture, and 13 had intraperitoneal rupture. Two children had metastatic disease at presentation.

One child died during the acute hemorrhagic episode before further oncologic therapy. Among the remaining 13 children, nine underwent emergency radical resection, two underwent partial hepatectomy for hemostasis, one achieved hemorrhage control with TAE, and one underwent salvage RFA after unsuccessful TAE. Eight of the 13 children who survived the acute event achieved complete response after first-line treatment, and three of these eight subsequently developed recurrence. By last follow-up, eight of the 14 children had died: one during the acute hemorrhagic episode and seven after documented progression or relapse (Table 2).

### 3.4. Survival

**Figure 3:**
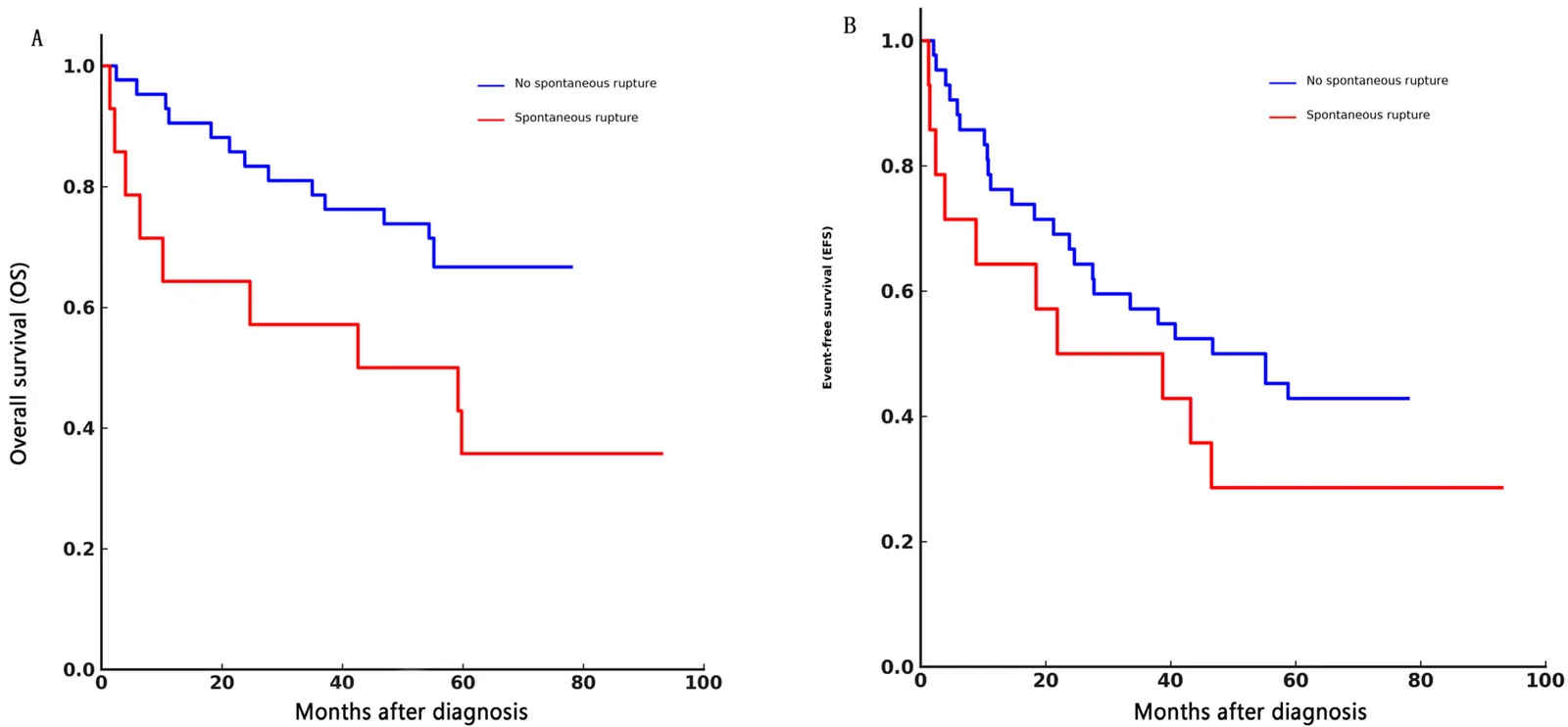
Kaplan–Meier curves for (**A**) overall survival and (**B**) event-free survival in the matched cohorts. EFS, event-free survival; OS, overall survival.

The median follow-up in the rupture cohort was 54.2 months (IQR 7.9–91.1). The rupture group had worse OS and EFS than the matched control group (Figure 3). The 1-, 3-, and 5-year OS rates were 64.8%, 54.1%, and 37.8% in the rupture group and 90.5%, 78.6%, and 66.7% in controls. Corresponding EFS rates were 62.2%, 46.6%, and 31.0% versus 85.7%, 73.8%, and 59.5%, respectively.

### 3.5. Exploratory Association and Model Analyses

Variables showing statistically significant between-group differences in the univariable analyses were entered into a conventional multivariable logistic regression model. In this model, greater maximum tumor diameter (OR 3.078, 95% CI 1.62–5.55; *p* < 0.001) and macrovascular invasion (OR 13.521, 95% CI 1.16–148.23; *p* = 0.037) remained associated with spontaneous rupture after adjustment, whereas tumor distribution and PRETEXT stage were no longer statistically significant (Table 3). However, because only 14 rupture events were available relative to the number of covariates, this model was susceptible to sparse-data bias, overfitting, and coefficient instability. In particular, the wide confidence interval for macrovascular invasion indicates substantial uncertainty in the magnitude of its adjusted association. Accordingly, these conventional multivariable results were regarded as exploratory rather than as confirmation of independent risk factors.

To examine whether the observed associations were directionally consistent when alternative analytical approaches were applied, additional exact and penalized analyses were performed. In the crude Fisher’s exact analysis of the matched cohort, macrovascular invasion was associated with spontaneous rupture (OR 8.00, 95% CI 2.05–31.16; *p* = 0.0027; Tables S3 and S4). In an exploratory two-predictor L2 ridge-penalized logistic model including maximum tumor diameter and macrovascular invasion, macrovascular invasion retained a positive association with rupture (adjusted OR 2.89, 95% bootstrap CI 1.27–7.93), whereas the per-centimeter maximum tumor-diameter estimate was positive but imprecise (adjusted OR 1.09, 95% bootstrap CI 0.92–1.20) (Table 4). The consistent direction of the association for macrovascular invasion across the conventional, exact, and ridge-penalized analyses provides supportive, although not confirmatory, evidence that macrovascular invasion may represent a clinically relevant indicator of rupture risk. Larger tumor size also remained an important warning feature on the basis of the marked group-level difference and the conventional multivariable result.

The combined model incorporating maximum tumor diameter and macrovascular invasion showed an apparent AUC of 0.930 and an optimism-corrected AUC of 0.924 after bootstrap internal validation (Figure S1), suggesting promising internal discrimination. At the archived operating point, the reported sensitivity and specificity were 92.9% and 81.0%, respectively; the corresponding post hoc Wilson 95% confidence intervals based on the matched-cohort denominators were 68.5–98.7% and 66.7–90.0%. Calibration was variable across predicted-risk groups and was interpreted cautiously because only 14 rupture events were available (Figure S2). Overall, although these complementary analyses do not establish confirmed independent risk factors, they do support macrovascular invasion and greater tumor burden as potential clinical warning features for spontaneous rupture.

## 4. Discussion

Tumor rupture may occur secondary to biopsy, trauma, or chemotherapy or may develop spontaneously without any identifiable cause. This propensity score-matched study focused specifically on spontaneous HB rupture. Rupture clustered with lower hemoglobin, a significantly larger maximum tumor diameter, bilobar involvement, advanced PRETEXT stage, and macrovascular invasion. Macrovascular invasion showed an exploratory association across crude Fisher’s exact test and ridge-penalized analyses, but the small number of rupture events and the absence of matched-set modeling preclude confirmation of independent risk factors. Children with rupture also had lower OS and EFS rates and substantial acute and disease-related mortality.

These findings are broadly consistent with, but more narrowly defined than, previous reports. Chang et al. identified a primary-tumor diameter >13.4 cm and vascular invasion as independent risk factors in a cohort that included spontaneous, traumatic, biopsy-related, and post-chemotherapy rupture [10]. By contrast, our exposure definition excluded rupture after trauma or treatment, reducing etiologic heterogeneity. Our results support large tumor burden and vascular involvement as warning features.

The prognostic literature remains mixed. Pondrom et al. reported 3-year EFS and OS of 49.6% and 68.2%, respectively, with several peritoneal progression or relapse events among children with ruptured HB [5]. Zhang et al., however, reported no deaths among nine children with rupture in a small, single-center cohort [6]. Differences in rupture definition, coexisting high-risk features, emergency stabilization, definitive treatment, and follow-up likely explain part of this variation. In our cohort, spontaneous rupture was accompanied by lower 5-year OS and EFS estimates, but the markedly larger tumors, higher PRETEXT stages, and more frequent macrovascular invasion in the rupture group prevent separation of the effect of rupture itself from the aggressive tumor phenotype that predisposes to rupture.

The association with tumor burden is clinically and biologically plausible. Rapid tumor expansion could increase intratumoral pressure, promote ischemia and necrosis, and thin the tumor capsule. In this cohort, the marked difference in maximum tumor diameter is an important descriptive finding and supports tumor size as a warning feature; however, the adjusted per-centimeter ridge estimate was imprecise. Macrovascular invasion may indicate locally aggressive growth, venous obstruction, congestion, and tissue friability. These mechanisms were not directly measured and remain hypotheses rather than demonstrated causal pathways.

The immediate clinical priority after rupture is hemodynamic stabilization and hemorrhage control. Emergency resection may be necessary in unstable children or when interventional radiology is unavailable or unsuccessful, whereas TAE can provide rapid control in selected anatomically suitable patients [11,12,13]. RFA is not standard treatment for ruptured HB; its single salvage use in this cohort should be interpreted as an exceptional rescue strategy rather than as evidence of comparative effectiveness [19]. Once bleeding is controlled, care should transition promptly to protocol-based chemotherapy and definitive resection or transplantation when indicated [20].

The findings may inform monitoring without supporting prophylactic invasive treatment. Children with very large tumors and macrovascular invasion may merit a lower threshold for monitored admission, serial abdominal examination, repeated hemoglobin and coagulation testing, advance preparation of blood products, and early multidisciplinary review involving pediatric oncology, hepatobiliary surgery, anesthesia, critical care, and interventional radiology. These measures aim to reduce delay in recognizing deterioration; they cannot be assumed to prevent the underlying biological event.

The exploratory combined model provides a more coherent interpretation of the archived ROC analysis. Maximum tumor diameter and macrovascular invasion jointly yielded an apparent AUC of 0.930 and an optimism-corrected AUC of 0.924. The sensitivity and specificity estimates at the archived operating point have wide Wilson intervals. Calibration was variable across predicted-risk groups. These limitations, together with the small number of events and lack of external validation, mean that the model is hypothesis-generating and not ready for clinical deployment.

This study has limitations. It was retrospective and conducted at a single tertiary referral center. Ten of the 106 screened children (9.4%) were excluded because clinicopathologic information or follow-up was incomplete; if missingness was related to referral, disease severity, or outcome, complete-case analysis could have introduced selection bias, and the direction of that bias cannot be determined. Propensity score matching used only prespecified demographic and anthropometric variables to avoid adjusting for candidate tumor features, but it did not balance tumor biology. Moreover, the matched-set structure was not incorporated into subsequent comparisons, regression, or survival summaries, so residual confounding and variance underestimation remained possible. Portal and hepatic venous involvement were combined because of sparse events, precluding vessel-specific estimates. Treatment practices and access to surgery, embolization, critical care, chemotherapy, and transplantation evolved during the 10-year study period; calendar-time effects were not formally modeled, and emergency treatment was individualized. Finally, internal bootstrap correction does not substitute for external validation. Multicenter studies using standardized rupture definitions, prospectively specified analyses, matched-set-aware methods, and calendar-period adjustment are needed.

## 5. Conclusions

Spontaneous hepatoblastoma rupture in children is associated with locally advanced tumor features and a low estimated long-term survival rate. Large tumor size and macrovascular invasion are considered potential risk factors for spontaneous HB rupture. Rupture should be regarded both as an emergency event and as a marker of biologically and clinically advanced disease. Early identification of high-risk imaging features, vigilant monitoring, and expedited multidisciplinary management may help reduce acute mortality and improve the chance of subsequent curative treatment.

## Figures and Tables

**Table 1:** Selected characteristics of the propensity score-matched cohorts at presentation.

Characteristic	Rupture Group (*n* = 14)	Matched Control Group (*n* = 42)	*p*-Value
Age, months	76.40 (29.48–120.20)	71.88 (26.40–118.52)	0.578
Male sex, *n* (%)	8 (57.1)	23 (54.8)	0.876
Weight, kg	20.15 (12.25–32.53)	21.31 (13.57–30.12)	0.147
Height, cm	111.00 (89.45–129.02)	107.63 (90.58–123.48)	0.364
Hemoglobin, g/L	80.00 (60.00–100.00)	103.00 (100.00–115.00)	0.032
Bilobar disease, *n* (%)	13 (92.9)	19 (45.2)	0.002
Maximum tumor diameter, cm	14.00 (10.88–15.45)	10.55 (8.31–12.15)	<0.001
PRETEXT stage III–IV, *n* (%)	13 (92.9)	19 (45.2)	0.002
Macrovascular invasion, *n* (%)	10 (71.4)	10 (23.8)	0.003

Values are median (IQR) unless otherwise indicated. Bilobar disease and PRETEXT III–IV *p*-values were recalculated from the displayed binary counts using Fisher’s exact tests. PRETEXT, pretreatment extent of disease.

**Table 2:** Presentation, emergency management, and outcomes of the spontaneous rupture cohort.

Patient	Sex/Age (m)	PRETEXT	Histopathology	AFP	Metastasis	Rupture Type	Tumor Site	Maximum Diameter of Tumor (cm)	Vascular Invasion	Treatment	CR at the End of First-Line Treatment	Event	Status at Last Follow-Up
1	M/5.1	II	Epi/Fet	885,312	No	Subcapsular	Lhl	10.1	No	RR	Yes	No	Alive (10.0 y)
2	M/14.2	III	Mix/Stro	47,923	No	Intraperitoneal	Lhl/Ra	10.9	Yes	RR	Yes	R	Death (5.0 y)
3	F/22.5	III	Epi/Em	12,715	No	Intraperitoneal	Rhl/Lm	10.4	No	RR	Yes	No	Alive, (7.9 y)
4	M/54.4	IV	Epi/Sma	412,455	Yes/AN	Intraperitoneal	Lhl/Rhl	15.8	Yes	PH	No	PD	Death (0.1 y)
5	F/78.3	III	Mix/Tera	507,831	No	Intraperitoneal	Rhl/Lm	15.0	Yes	RR	Yes	R	Alive (11.0 y)
6	F/31.8	III	Mix/Stro	15,002	No	Intraperitoneal	Rhl/Lm	10.9	No	RR	Yes	No	Alive (6.3 y)
7	M/89.1	IV	Epi/Em	44,000	Yes/Lung	Intraperitoneal	Lhl/Rhl	13.0	Yes	TAE	No	PD	Death (0.8 y)
8	M/56.1	III	Uncertain	361,789	No	Intraperitoneal	Rhl/Lm	12.5	Yes	CT	No	PD	Death (0 y)
9	M/74.5	III	Epi/Fet	808,315	No	Intraperitoneal	Rhl/Lm	11.2	No	RR	Yes	No	Alive (5.6 y)
10	F/105.0	IV	Mix/Stro	842,167	No	Intraperitoneal	Lhl/Rhl	15.2	Yes	PH	No	PD	Death (0.7 y)
11	M/141.4	III	Epi/Fet	145,219	No	Intraperitoneal	Rhl/Lm	15.6	Yes	RR	No	PD	Death (2.6 y)
12	F/118.1	III	Epi/Em	781,455	No	Intraperitoneal	Lhl/Ra	15.1	Yes	RR	Yes	R	Death (4.1 y)
13	F/126.5	III	Mix/Tera	341,123	No	Intraperitoneal	Rhl/Lm	15.4	Yes	RR	Yes	No	Alive (7.5 y)
14	M/132.3	IV	Epi/Fet	27,851	No	Intraperitoneal	Lhl/Rhl	16.0	Yes	RFA	No	PD	Death (0.6 y)

Epi, epithelial; Fet, fetal; Em, embryonal; Sma, small-cell undifferentiated; Mix, mixed; Stro, stromal derivatives; Tera, teratoid; AN, abdominal node; Lhl, left hepatic lobe; Rhl, right hepatic lobe; Ra, right anterior section; Lm, left medial section; RR, radical resection; PH, partial hepatectomy; TAE, transcatheter arterial embolization; CT, conservative treatment; RFA, radiofrequency ablation; CR, complete response; R, relapse; PD, progressive disease.

**Table 3:** Risk factors for spontaneous rupture of hepatoblastoma.

Variables	Univariate Analysis	Multivariable Analysis		
	*p*	OR	95%CI	*p*
Maximum diameter of tumor	<0.001	3.078	1.62–5.55	<0.001
Macrovascular invasion	0.003	13.521	1.16–148.23	0.037
Tumor site (unilobar or bilobar)	0.024	1.211	0.75–2.21	0.621
PRETEXT (III–IV or I–II)	0.019	1.006	0.21–2.15	0.422

**Table 4:** Exploratory association and internal model-performance analyses for spontaneous rupture of hepatoblastoma.

Analysis	Variable or Metric	Estimate	95% CI	*p*-Value or Interpretation
Fisher’s exact test	Macrovascular invasion (yes vs. no)	Crude OR 8.00	2.05–31.16	*p* = 0.0027
Ridge-penalized logistic regression	Maximum tumor diameter (per 1 cm)	Adjusted OR 1.09	0.92–1.20	2000-bootstrap percentile CI
Ridge-penalized logistic regression	Macrovascular invasion (yes vs. no)	Adjusted OR 2.89	1.27–7.93	2000-bootstrap percentile CI
Combined-model discrimination	Apparent AUC	0.930	Not available	Exploratory
Bootstrap internal validation	Optimism-corrected AUC	0.924	Not available	2000 resamples
Archived operating point	Sensitivity	92.9%	68.5–98.7%	Wilson 95% CI; threshold unavailable
Archived operating point	Specificity	81.0%	66.7–90.0%	Wilson 95% CI; threshold unavailable

Sensitivity and specificity confidence intervals were calculated by the Wilson score method from the archived classification counts.

## Data Availability

The data are not publicly available because they contain potentially identifiable pediatric clinical information and are subject to institutional privacy restrictions. De-identified data may be available from the corresponding author upon reasonable request, subject to approval by the institutional ethics committee and an appropriate data-use agreement.

## Supplementary Materials

The following supporting information can be downloaded at https://www.mdpi.com/article/10.3390/jcm15187102/s1, Figure S1: Receiver operating characteristic curve for the exploratory combined model; Figure S2: Calibration plot for the exploratory combined model; Table S1: Baseline demographic balance before and after propensity score matching; Table S2: Full clinical characteristics at initial presentation; Table S3: Contingency table for macrovascular invasion and spontaneous rupture; Table S4: Fisher’s exact sensitivity analysis.
