# Supplementary material for: Clinical Risk Factors and Outcomes of Spontaneous Hepatoblastoma Rupture in Children: A 10-Year Propensity Score-Matched Study
Source: J Clin Med. 2026 Sep 13;15(18):7102. doi: 10.3390/jcm15187102 (PMC13607828; doi:10.3390/jcm15187102)
Supplement: Supplementary file 1 [file jcm-15-07102-s001.zip › Table S1.pdf]

Table S1. Baseline demographic balance before and after propensity score matching.

Characteristic	Before matching		SMD	P	After matching		SMD	P
	Tumour rupture (n=14)	Non-tumour rupture (n=82)			Tumour rupture (n=14)	Non-tumour rupture (n=42)		
Age, m	76.40(29.48,120.20)	59.25(23.00,106.75)	0.382	0.086	76.40(29.48,120.20)	71.88 (26.40, 118.52)	0.064	0.578
Gender, n(%)								
male	8(57.1)	45(54.9)	0.044	0.871	8(57.1)	23(54.8)	0.048	0.876
female	6(42.9)	37(45.1)			6(42.9)	19(45.2)		
Weight, kg	20.15(12.25,32.53)	16.22(11.25,27.72)	0.794	0.029	20.15(12.25,32.53)	21.31 (13.57, 30.12)	0.098	0.147
Height, cm	111.00(89.45,129.02)	100.75(81.25,121.49)	0.687	0.032	111.00(89.45,129.02)	107.63 (90.58, 123.48)	0.075	0.364

Values are median (IQR) unless otherwise indicated. SMD, standardized mean difference.
