# Supplementary material for: Clinical Risk Factors and Outcomes of Spontaneous Hepatoblastoma Rupture in Children: A 10-Year Propensity Score-Matched Study
Source: J Clin Med. 2026 Sep 13;15(18):7102. doi: 10.3390/jcm15187102 (PMC13607828; doi:10.3390/jcm15187102)
Supplement: Supplementary file 1 [file jcm-15-07102-s001.zip › Table S2.pdf]

Table S2. Full clinical characteristics at initial presentation.

Characteristic	Rupture group(n=14)	Control group(n=42)	P
Gender, n(%)			0.876
Male	8(57.1)	23(54.8)	
Female	6(42.9)	19(45.2)	
Age, m	76.40(29.48,120.20)	71.88 (26.40, 118.52)	0.578
Weight, kg	20.15(12.25,32.53)	21.31 (13.57, 30.12)	0.147
Height, cm	111.00(89.45,129.02)	107.63 (90.58, 123.48)	0.364
Blood type, n (%)			0.287
A	5（35.7）	10（23.8）	
B	2（14.3）	10（23.8）	
AB	5（35.7）	13（31.0）	
O	2（14.3）	9（21.4）	
Alpha-fetoprotein, ng/ml	351,456（44,000,781,455）	392,711（42,817,801,741）	0.740
Hemoglobin, g/L	80.00（60.00，100.00）	103.00（100.00，115.00）	0.032
White blood cell count, 10^9/L	10.61（9.19，14.31）	10.47（7.54，12.08）	0.363
Platelet count, 10^9/dL	167.00（76.33）	175.55（75.22）	0.897
Alanine aminotransferase, U/L	20.00（16.00，29.00）	24.50（18.00，39.75）	0.519
Aspartate aminotransferase, U/L	22.00（13.00，39.75）	27.00（15.00，42.75）	0.430
Alkaline phosphatase, U/L	76.50（57.00，88.75）	80.00（61.00，98.15）	0.350
γ-glutamyl transferase, U/L	17.00（10.75，39.25）	19.55（12.00，37.50）	0.711
Total bilirubin, μmol	8.20（2.44，10.90）	7.30（2.85，9.65）	0.756
Direct bilirubin, μmol	3.02（1.32，4.90）	3.30（1.85，4.65）	0.580

Albumin, g/L	40.40（28.65，44.85）	46.90（42.13，48.30）	0.052
Serum creatinine, μmol	18.00（14.00，26.75）	21.00（15.23，34.08）	0.101
Blood urea nitrogen, mmol/L	4.08（3.20，5.53）	4.25（3.35，5.68）	0.102
International normalized ratio	1.40（1.14，1.67）	1.26（1.09，1.53）	0.291
Child-Pugh Grade，n(%)			0.612
A	13（92.9）	35（83.3）	
B	1（7.1）	7（16.7）	
C	0（0）	0（0）	
Tumor site, n(%)			0.024
Left hepatic lobe	1（7.1）	15(35.7)	
Right hepatic lobe	0（0）	8(19.1)	
Left hepatic lobe/right anterior section	2（14.3）	6(14.2)	
Right hepatic lobe/left medial section	7（50.0）	8(19.1)	
Both left and right hepatic lobes are involved	4（28.6）	5(11.9)	
Maximum diameter of primary tumor, cm	14.00（10.88，15.45）	10.55（8.31，12.15）	＜0.001
Histopathology, n (%)			0.938
Epithelial variants	8（57.2）	24（57.1）	
Mixed epithelial and mesenchymal	5（35.7）	16（38.1）	
Uncertain	1（7.1）	2（4.8）	

PRETEXT staging, n (%)			0.019
I	0（0）	8（19.1）	
II	1（7.1）	15（35.7）	
III	9（64.3）	14（33.3）	
IV	4（28.6）	5（11.9）	
Macrovascular invasion, n (%)			0.003
Yes	10（71.4）	10（23.8）	
No	4（28.6）	32（76.2）	

Values are median (interquartile range) unless otherwise indicated. Laboratory data were obtained at initial presentation before emergency intervention or chemotherapy.
